# Long-Term Outcome of West Syndrome Treated With Adrenocorticotropic Hormone-Based Therapy in a Tertiary Care Hospital in Bangladesh

**DOI:** 10.7759/cureus.117115

**Published:** 2026-09-28

**Authors:** Samsun N Sumi, Abdullah A Mamun, Sufia K Sumi, Sarah Alam, Ahsan Habib, Mosiur Rahman, Nazmul Haque, Tuhfie Jannat, Yamin Shahriar Chowdhury, Rajib N Chowdhury

**Affiliations:** 1 Department of Pediatric Neurology, National Institute of Neurosciences & Hospital, Dhaka, BGD; 2 Department of Neurophysiology, National Institute of Neurosciences & Hospital, Dhaka, BGD; 3 Department of Colorectal Surgery, Dhaka Medical College & Hospital, Dhaka, BGD; 4 Department of Pediatric Neurology, Khulna Medical College &amp; Hospital, Khulna, BGD; 5 Department of Neurology, National Institute of Neurosciences & Hospital, Dhaka, BGD

**Keywords:** acth therapy, developmental outcome, eeg, lennox-gastaut syndrome, seizure outcome, vigabatrin, west syndrome

## Abstract

Background

West syndrome is a severe developmental and epileptic encephalopathy associated with epileptic spasms, abnormal EEG findings, and poor long-term neurodevelopmental outcomes. Adrenocorticotropic hormone (ACTH)-based therapies are widely used for treatment, but data on seizure, EEG, and developmental outcomes from Bangladesh remain limited.

Objective

The aim of this study was to evaluate seizure outcomes, EEG changes, and long-term functional developmental outcomes among children with West syndrome treated with different ACTH-based regimens in a tertiary care hospital in Bangladesh.

Methods

This retrospective observational follow-up study included 50 children with West syndrome treated at the Department of Pediatric Neurology, National Institute of Neurosciences & Hospital, Dhaka, Bangladesh, between 2018 and 2024. Children were categorized into three treatment groups: high-dose ACTH, n = 18; combination therapy with high-dose ACTH plus vigabatrin, n = 10; and medium-dose ACTH, n = 22. Seizure outcomes, post-treatment EEG findings, and long-term functional developmental outcomes were assessed from clinical records, EEG reports, caregiver interviews, and structured parental surveys. Data were analyzed using SPSS Version 22.0, with p < 0.05 considered statistically significant.

Results

Baseline age distribution, age at onset of spasms, and sex did not differ significantly among treatment groups. Seizure outcome differed significantly across groups, with seizure freedom observed in 61.1% of children in the high-dose ACTH group, 50.0% in the combination therapy group, and 4.5% in the medium-dose ACTH group. Lennox-Gastaut syndrome was most frequent in the medium-dose ACTH group, 72.7%. EEG normalization was highest in the high-dose ACTH group, 44.4%, followed by the combination therapy group, 20.0%, and the medium-dose ACTH group, 13.6%, although this difference was not statistically significant. Developmental outcome also did not differ significantly among groups; however, dependent living was most frequent in the medium-dose ACTH group, 59.1%.

Conclusion

High-dose ACTH was associated with better seizure freedom and a higher proportion of EEG normalization in this cohort, while medium-dose ACTH was associated with poorer seizure outcome, more frequent Lennox-Gastaut syndrome, and greater developmental dependency. However, the retrospective non-randomized design and small sample size limit causal interpretation. Larger prospective studies with standardized treatment protocols, etiological classification, relapse assessment, adverse-effect monitoring, and validated developmental evaluation are needed.

## Introduction

West syndrome (WS) is a rare yet severe pediatric epileptic encephalopathy, predominantly manifesting within the first year of life. It is clinically characterized by a triad of infantile spasms, hypsarrhythmia on electroencephalogram (EEG), and developmental regression, which together delineate its devastating impact on neurodevelopmental outcomes [1]. The global prevalence of WS is estimated to range from approximately 1 in 2,000 to 6,000 live births, though accurate data remain elusive due to underreporting, particularly in resource-constrained settings [2]. This paucity of data is even more pronounced in developing countries like Bangladesh, where epidemiological studies on WS remain scarce, underscoring the urgent need for region-specific research.

The disorder imposes a profound burden not only on the affected children but also on their families, amplifying the socioeconomic challenges inherent in low-resource healthcare systems [3]. The etiology of WS is heterogeneous, encompassing genetic, structural, and idiopathic causes. Genetic mutations, such as those in the ALG13 gene, have been increasingly implicated in the pathogenesis of WS, alongside structural abnormalities such as hypoxic-ischemic encephalopathy [4]. Idiopathic WS, where no underlying cause is identified, tends to have a relatively favorable prognosis compared to symptomatic forms [5]. Despite advancements in genetic testing and neuroimaging, diagnostic challenges persist, particularly in developing regions where access to advanced diagnostic tools is limited [6]. Early and accurate diagnosis is pivotal, as delayed intervention often correlates with poorer neurodevelopmental outcomes and higher risks of comorbid epilepsy [7].

Adrenocorticotropic hormone (ACTH) therapy remains the cornerstone treatment for WS, demonstrating superior efficacy in controlling infantile spasms compared to other interventions such as vigabatrin or oral corticosteroids [8]. ACTH exerts its therapeutic effects through modulation of the hypothalamic-pituitary-adrenal axis, increasing cortisol levels and stabilizing neuronal excitability. Its immunomodulatory actions, including the suppression of pro-inflammatory cytokines, further contribute to its anticonvulsant properties [9]. Studies have shown that both high-dose and low-dose ACTH regimens can be effective, with low-dose protocols offering comparable efficacy while minimizing adverse effects such as hypertension, infections, and brain shrinkage [10,11]. Additionally, long-term ACTH therapy has shown promise in managing relapsed WS, particularly in symptomatic cases, though its utility remains constrained by potential toxicities and high relapse rates [12]. Despite its clinical benefits, ACTH therapy is not without challenges. Access to ACTH is limited in many developing regions due to high costs and logistical hurdles, exacerbating disparities in WS management [6]. Furthermore, the lack of uniform guidelines for ACTH dosing and treatment duration adds to the variability in therapeutic outcomes [8]. Comparative studies between ACTH and alternative therapies, such as methylprednisolone, suggest that the latter may offer similar efficacy with a reduced side-effect profile, presenting a viable option in resource-constrained settings [13]. However, ACTH remains the preferred first-line therapy in most guidelines due to its robust evidence base. In Bangladesh, the absence of epidemiological data and resource-specific treatment protocols for WS compounds the challenges faced by healthcare providers. This study aims to evaluate the efficacy of ACTH-based therapies in managing WS within the Bangladeshi context, focusing on both clinical outcomes and the socioeconomic implications of treatment. By addressing these gaps, the findings aim to contribute to the optimization of WS management strategies in resource-constrained settings, ultimately improving neurodevelopmental outcomes and quality of life for affected children and their families.

## Materials and methods

Study design and setting

This single-center retrospective observational follow-up study was conducted at the Department of Pediatric Neurology, National Institute of Neurosciences & Hospital, Dhaka, Bangladesh. The study included children diagnosed with WS who received ACTH-based therapy between 2018 and 2024 and had available follow-up data after completion of treatment. WS was diagnosed based on the presence of epileptic spasms with EEG evidence of classical or modified hypsarrhythmia.

Study participants and eligibility criteria

Children were included if they had a confirmed diagnosis of WS, documented treatment with one of the ACTH-based regimens, available baseline clinical information, available post-treatment seizure and EEG outcome data, and adequate long-term follow-up information. Children were excluded if they had incomplete treatment records, inadequate baseline documentation, unavailable seizure or EEG outcome data, or insufficient follow-up information. A total of 50 children fulfilled the eligibility criteria and were included in the final analysis. Eligibility was assessed using available clinical records rather than a pre-specified structured screening algorithm; this reliance on documented clinical judgment is acknowledged as a potential source of selection variability (see the Limitations section).

Treatment regimens

Participants were categorized into three treatment groups according to the regimen received: high-dose ACTH, n = 18; combination therapy with high-dose ACTH plus vigabatrin, n = 10; and medium-dose ACTH, n = 22. In the high-dose ACTH group, natural ACTH was administered intramuscularly at 150 IU/m²/day in two divided doses for 14 days, followed by tapering; the subsequent taper was individualized by the treating physician according to clinical response rather than a fixed protocolized schedule, and the exact taper duration was not uniformly documented for separate reporting. In the combination therapy group, the same high-dose ACTH protocol was used together with oral vigabatrin, which was titrated to 100-150 mg/kg/day, continued for three months, and tapered over four weeks. In the medium-dose ACTH group, ACTH was administered at 110 IU/m²/day for one month, followed by alternate-day dosing for one month and twice-weekly dosing for another month. Treatment allocation was not randomized and was based on clinical decision-making, patient condition, drug availability, and treatment feasibility, without a pre-specified decision algorithm; therefore, comparisons between treatment groups were interpreted as exploratory rather than confirmatory evidence of treatment superiority, and exact replication of treatment-group assignment by other investigators may not be possible.

Data collection and outcome measures

Data were collected from hospital records, EEG reports, clinical follow-up notes, caregiver interviews, and structured parental surveys. Baseline variables included current age, age at onset of spasms, sex, pre-spasm developmental status, family history of epilepsy, history of consanguinity, treatment regimen, and current antiseizure medication use. Current age was defined as the child's age at the time of the most recent post-treatment clinical or follow-up assessment used for this analysis, distinct from age at onset of spasms. The current-age categories (≤50, 51-60, and >60 months) were descriptive groupings based on the observed age distribution in this cohort, used only to assess baseline comparability, and were not used for outcome stratification; based on these categories, current age across the cohort ranged from ≤50 months to more than 60 months. The interval between onset of spasms and initiation of ACTH-based treatment was not consistently documented in the available records and could not be analyzed; this is acknowledged as a limitation. Follow-up variables included seizure status, evolution into other seizure types or epileptic syndromes, post-treatment EEG findings, current medication use, and functional developmental outcome. The primary outcome was seizure outcome following ACTH-based therapy, categorized as seizure-free, focal epilepsy, Lennox-Gastaut syndrome, generalized tonic-clonic seizures, or myoclonic epilepsy. This primary outcome combined seizure-free status, seizure type, and epilepsy syndrome (Lennox-Gastaut syndrome) into a single descriptive categorical variable for the purposes of this analysis; these categories are conceptually heterogeneous, and this simplification is acknowledged as a limitation of the outcome framework used. Lennox-Gastaut syndrome was diagnosed based on clinical seizure pattern, developmental status, and EEG findings compatible with epileptic encephalopathy. Secondary outcomes included post-treatment EEG findings and long-term functional developmental outcome. Post-treatment EEG findings were categorized as normal EEG, focal epileptiform discharge, generalized epileptiform discharge, or epileptic encephalopathy. EEG interpretation for this analysis was based on the original written reports issued by the reporting neurophysiology or pediatric neurology specialist using the most recent available post-treatment EEG record; EEG recordings were not independently re-reviewed in a blinded fashion by the study authors, which may introduce inter-rater variability and is acknowledged as a limitation. Long-term functional developmental outcome was assessed at least two years after completion of ACTH-based therapy using clinical evaluation, caregiver interview, structured parental survey, and the Independent Behavior Assessment Scale for children aged two to nine years [14]. This minimum two-year post-treatment interval was applied uniformly across all treatment groups; however, the exact follow-up duration beyond this minimum threshold was not systematically recorded or statistically compared between groups, and unequal follow-up duration could have influenced the observed seizure, EEG, and developmental outcomes. Functional outcome was categorized into normal living, assisted living, and dependent living groups. The normal living group included children with age-appropriate or near age-appropriate functional independence in daily activities, the assisted living group included children requiring partial caregiver assistance for daily functioning, and the dependent living group included children requiring substantial or full caregiver support for daily living activities. These functional living categories reflect the degree of caregiver support required for daily activities and were not designed to capture domain-specific neurodevelopmental status; a child classified as achieving normal or assisted living could still have unrecognized intellectual, language, or behavioral impairment. Accordingly, these categories should not be equated with normal neurodevelopment, and this is acknowledged as a limitation of the outcome measure used.

Statistical analysis

Data were analyzed using SPSS version 22.0. Continuous variables were expressed as mean ± standard deviation, and categorical variables were expressed as frequency and percentage. Comparisons among the three treatment groups were performed using one-way analysis of variance or Kruskal-Wallis test for continuous variables, depending on data distribution. Categorical variables were compared using the chi-square test or Fisher's exact test, as appropriate, with Fisher's exact test considered when expected cell counts were small. A two-sided p-value of <0.05 was considered statistically significant. Because the study was retrospective, non-randomized, and included small treatment subgroups, statistical comparisons were interpreted cautiously as exploratory and hypothesis-generating.

Ethical considerations

The study was conducted in accordance with the principles of the Declaration of Helsinki. Ethical approval was obtained from the local institutional ethics committee. Written informed consent was obtained from parents or legal guardians before follow-up assessment and caregiver interview. Patient confidentiality was maintained throughout data collection, analysis, and reporting.

## Results

A total of 50 children with WS treated with ACTH-based regimens were included in the final analysis. Baseline demographic and clinical characteristics of the study participants are presented in Table 1.

**Table 1:** Baseline characteristics of study subjects (N=50) Values are n (%) unless otherwise indicated. Age category (≤50, 51–60, >60 months) was compared using Fisher's exact test. Age at onset of spasms (mean ± SD) was compared using one-way ANOVA, F(2,47) = 2.24. Sex was compared using Pearson's chi-square test, χ²(2) = 4.70. ACTH, adrenocorticotropic hormone

Characteristic	High-dose ACTH (n=18)	Combination therapy (n=10)	Medium-dose ACTH (n=22)	p-Value
Age				
Age (months) ≤50, n (%)	3 (16.7%)	3 (30.0%)	5 (22.7%)	0.789
Age (months) 51-60, n (%)	8 (44.4%)	6 (60.0%)	12 (54.5%)	
Age (months) >60, n (%)	7 (38.9%)	1 (10.0%)	5 (22.7%)	
Age of onset of spasms (mean ± SD, months)	7.6 ± 3.6	5.5 ± 2.1	6.0 ± 2.5	0.133
Gender				
Male, n (%)	12 (66.7%)	3 (30.0%)	15 (68.2%)	0.096
Female, n (%)	6 (33.3%)	7 (70.0%)	7 (31.8%)	

Baseline characteristics did not differ significantly among the three treatment groups for any measured variable, including current-age distribution (p=0.789), age at onset of spasms (p=0.133), and sex (p=0.096), indicating that the groups were broadly comparable at baseline (Table 1). Seizure outcomes among the three treatment groups are presented in Table 2.

**Table 2:** Seizure outcomes (N=50) Values are presented as n (%). Seizure outcome was compared using Pearson's chi-square test, χ²(8) = 20.35. ACTH, adrenocorticotropic hormone; LGS, Lennox-Gastaut syndrome; GTCS, generalized tonic-clonic seizures

Outcome of seizure	High-dose ACTH (n=18), n (%)	Combination therapy (n=10), n (%)	Medium-dose ACTH (n=22), n (%)	p-Value
Seizure-free	11 (61.1%)	5 (50.0%)	1 (4.5%)	0.009
Focal epilepsy	2 (11.1%)	1 (10.0%)	2 (9.1%)	
LGS	3 (16.7%)	3 (30.0%)	16 (72.7%)	
GTCS	1 (5.6%)	0 (0.0%)	0 (0.0%)	
Myoclonic epilepsy	1 (5.6%)	1 (10.0%)	3 (13.6%)	

Seizure outcome differed significantly across the three treatment groups (p=0.009). The high-dose ACTH group had the highest seizure-free rate (11, 61.1%), followed by the combination therapy group (5, 50.0%), while only one (4.5%) child in the medium-dose ACTH group achieved seizure freedom. Lennox-Gastaut syndrome was markedly more frequent in the medium-dose ACTH group (16, 72.7%) than in the combination therapy (3, 30.0%) or high-dose ACTH groups (3, 16.7%); focal epilepsy, generalized tonic-clonic seizures, and myoclonic epilepsy were uncommon across all groups (Table 2). Post-treatment EEG findings across the treatment groups are presented in Table 3.

**Table 3:** Change in EEG findings (N=50) Values are presented as n (%). Change in EEG findings was compared using Pearson's chi-square test, χ²(6) = 8.48. ACTH, adrenocorticotropic hormone

Change in EEG	High-dose ACTH (n=18), n (%)	Combination therapy (n=10), n (%)	Medium-dose ACTH (n=22), n (%)	p-Value
Normal	8 (44.4%)	2 (20.0%)	3 (13.6%)	0.205
Focal epileptiform discharge	4 (22.2%)	4 (40.0%)	12 (54.5%)	
Generalized epileptiform discharge	3 (16.7%)	1 (10.0%)	1 (4.5%)	
Epileptic encephalopathy	3 (16.7%)	3 (30.0%)	6 (27.3%)	

Post-treatment EEG findings showed clinically notable but statistically non-significant differences among groups (p=0.205). Normal EEG was most frequent in the high-dose ACTH group (8, 44.4%), while focal epileptiform discharge was most frequent in the medium-dose ACTH group (12, 54.5%); generalized epileptiform discharge and epileptic encephalopathy occurred at broadly similar, low rates across groups (Table 3). Long-term functional developmental outcomes for the study participants are presented in Table 4.

**Table 4:** Long-term functional developmental outcomes Values are presented as n (%). Developmental outcome was compared using Pearson's chi-square test, χ²(4) = 1.96. ACTH, adrenocorticotropic hormone

Outcome of development	High-dose ACTH (n=18), n (%)	Combination therapy (n=10), n (%)	Medium-dose ACTH (n=22), n (%)	p-Value
Normal living group	5 (27.8%)	3 (30.0%)	3 (13.6%)	0.744
Assisted living group	5 (27.8%)	3 (30.0%)	6 (27.3%)	
Dependent living group	8 (44.4%)	4 (40.0%)	13 (59.1%)	

Developmental outcome did not differ significantly among treatment groups (χ² test, p=0.744). Dependent living was the most common outcome overall and was most frequent in the medium-dose ACTH group (13, 59.1%), while normal living was more frequent in the high-dose ACTH and combination therapy groups (Table 4).

## Discussion

The present study evaluated seizure outcomes, EEG changes, and developmental outcomes among children with WS treated with ACTH-based regimens in a tertiary care hospital in Bangladesh. Baseline characteristics, including age distribution, age at onset of spasms, and sex, did not differ significantly among the three treatment groups. This suggests that the groups were broadly comparable for the measured demographic variables, consistent with previous studies where age of onset and sex distribution showed limited variation among children with WS [15,16]. The mean age of spasm onset in this cohort ranged from 5.5 to 7.6 months, which is similar to the commonly reported onset during early infancy [17]. Male predominance in the high-dose and medium-dose ACTH groups was also in line with the reported male preponderance in WS [18].

Seizure outcome showed the clearest difference among treatment groups. The seizure-free rate was highest in the high-dose ACTH group (11, 61.1%), followed by the combination therapy group (5, 50.0%), whereas only 1 (4.5%) of children in the medium-dose ACTH group became seizure-free. This difference was statistically significant, suggesting a more favorable seizure-control profile associated with high-dose ACTH-based treatment in this cohort. This finding is consistent with previous evidence supporting ACTH as an effective therapy for infantile spasms, particularly when adequate dosing is used [8]. However, because this was a retrospective and non-randomized study, the observed difference should be interpreted cautiously. Treatment response may have been influenced by unmeasured factors such as etiology, treatment delay, baseline EEG severity, neuroimaging abnormalities, and pre-existing developmental status.

The pattern of later seizure evolution also deserves attention. Lennox-Gastaut syndrome was most frequent in the medium-dose ACTH group, affecting 16 (72.7%) of children, compared with 3 (30.0%) in the combination therapy group and 3 (16.7%) in the high-dose ACTH group. This suggests that children in the medium-dose group had a higher burden of persistent epileptic encephalopathy or later epileptic evolution. The poorer seizure-free rate in this group raises the possibility of a dose-response relationship, although this remains uncertain and requires further study [19]. The high frequency of Lennox-Gastaut syndrome in the medium-dose group is clinically important because progression from WS to Lennox-Gastaut syndrome is associated with difficult-to-control seizures and poorer long-term neurodevelopmental outcomes, supporting the importance of effective early treatment [20].

EEG changes showed clinically relevant trends, although the differences were not statistically significant. Normal EEG findings were most frequent in the high-dose ACTH group (8, 44.4%), followed by the combination therapy group (2, 20.0%), and the medium-dose ACTH group (3, 13.6%). This pattern was consistent with the seizure outcome findings, as the group with the highest seizure-free rate also showed the highest proportion of EEG normalization. Previous studies have emphasized the prognostic importance of EEG improvement in WS [21]. In contrast, persistent focal epileptiform discharges were most common in the medium-dose ACTH group (12, 54.5%), compared with four (40.0%) in the combination therapy group and four (22.2%) in the high-dose ACTH group. This persistence of epileptiform activity may reflect ongoing cortical irritability, incomplete electroclinical remission, or underlying structural pathology, similar to observations reported in previous EEG outcome studies [22].

Epileptic encephalopathy on follow-up EEG was observed in all groups, including three (16.7%) of the high-dose ACTH group, three (30.0%) of the combination therapy group, and six (27.3%) of the medium-dose ACTH group. These findings highlight that even after ACTH-based treatment, a substantial proportion of children continue to have abnormal EEG activity. Such persistence may be related to underlying structural, genetic, or metabolic etiologies, which are difficult to fully address through antiseizure therapy alone [6]. Therefore, clinical seizure control should not be the only marker of treatment response; follow-up EEG remains important for identifying persistent epileptic encephalopathy and ongoing relapse risk.

Developmental outcome remained poor across all groups. Although the difference was not statistically significant, normal living was more frequent in the combination therapy group (3, 30.0%) and high-dose ACTH group (5, 27.8%) than in the medium-dose ACTH group (3, 13.6%). Conversely, dependent living was most frequent in the medium-dose group (13, 59.1%), compared with 8 (44.4%) in the high-dose ACTH group and 4 (40.0%) in the combination therapy group. These findings suggest that poorer seizure control and persistent EEG abnormalities may be associated with worse functional outcome. However, developmental outcome in WS depends on multiple factors, including etiology, baseline developmental delay, treatment lag, relapse, and access to rehabilitation. Thus, seizure freedom alone may not ensure normal development.

Overall, the findings support the role of ACTH-based therapy as a cornerstone treatment for WS while also showing that long-term outcomes remain heterogeneous [8,23]. Although not systematically measured in this study, contextual factors previously described in the region, including delayed diagnosis, limited EEG availability, cost of ACTH, follow-up gaps, and restricted rehabilitation services may further influence outcomes and warrant direct assessment in future prospective studies. These challenges are consistent with regional observations regarding WS management in South Asia [18]. Future studies should include larger prospective cohorts, standardized treatment protocols, baseline and follow-up EEG comparison, etiological classification, relapse data, adverse-effect monitoring, and validated developmental assessment to better define predictors of long-term outcome. A more granular outcome framework that separately captures early treatment response, electroclinical response, relapse, long-term epilepsy, syndrome evolution, and developmental outcomes, rather than a single composite seizure-outcome category, would further strengthen future analyses.

Limitations of the study

This study has several limitations. Given the retrospective, non-randomized, single-center design and small, unequal subgroup sizes, this study is best interpreted as an exploratory descriptive analysis of outcome patterns associated with three locally used ACTH-based treatment pathways, rather than a confirmatory comparative-effectiveness study; the apparent advantage of high-dose ACTH should not be interpreted as an established causal treatment effect. It was conducted in a single tertiary care hospital with a small sample size and unequal treatment groups, limiting generalizability and statistical power. Treatment allocation was not randomized and did not follow a pre-specified decision algorithm; therefore, observed differences in seizure, EEG, and developmental outcomes may have been influenced by baseline disease severity, treatment delay, etiology, physician preference, drug availability, or parental affordability. Important prognostic variables, including neuroimaging findings, etiological classification, baseline EEG severity, relapse, adverse effects, and detailed rehabilitation history, were not fully analyzed. Developmental outcome was assessed using broad functional categories, which may not capture the full range of cognitive, motor, behavioral, and psychosocial impairment, and should not be equated with normal neurodevelopment. The interval between spasm onset and treatment initiation, the precise tapering schedule for the high-dose ACTH group, and follow-up duration beyond the two-year minimum were not systematically available for statistical comparison. EEG interpretation relied on original clinical reports rather than independent, blinded re-review by the study authors, and the primary seizure-outcome variable combined outcome status, seizure type, and epilepsy syndrome into a single descriptive category; both are acknowledged as methodological limitations.

## Conclusions

This study showed that children with WS treated with ACTH-based regimens had variable long-term seizure, EEG, and developmental outcomes. High-dose ACTH was associated with the highest seizure-free rate and the greatest proportion of EEG normalization, while the medium-dose ACTH group showed the lowest seizure-free rate, the highest frequency of Lennox-Gastaut Syndrome, more persistent focal epileptiform discharges, and a greater proportion of dependent living. However, because the study was retrospective, non-randomized, and based on a small sample, these findings should be interpreted as exploratory rather than definitive evidence of treatment superiority. Developmental dependency remained common across all treatment groups, indicating that seizure control alone may not ensure favorable long-term neurodevelopmental outcomes. Early diagnosis, appropriate ACTH-based treatment, repeat EEG monitoring, relapse surveillance, and long-term multidisciplinary developmental support are essential for improving outcomes in children with WS in resource-limited settings.

## References

[1] Pavone P, Polizzi A, Marino SD, Corsello G, Falsaperla R, Marino S, Ruggieri M (2020). West syndrome: a comprehensive review. Neurol Sci.

[2] Taghdiri MM, Nemati H (2014). Infantile spasm: a review article. Iran J Child Neurol.

[3] Nguefack S, Moifo B, Chiabi A (2013). Clinical, aetiological and evolutive aspects of West syndrome in Yaoundé (Cameroon). Open J Pediatr.

[4] Madaan P, Negi S, Sharma R, Kaur A, Sahu JK (2019). X-linked ALG13 gene variant as a cause of epileptic encephalopathy in girls. Indian J Pediatr.

[5] Vigevano F, Fusco L, Cusmai R, Claps D, Ricci S, Milani L (1993). The idiopathic form of West syndrome. Epilepsia.

[6] Fogarasi A, Erdélyi F, Siegle Z (2018). Challenges in the treatment of West syndrome. Biomed J Sci & Tech Res.

[7] Chandrasekharan SV, Menon RN, Nanda S, Nair JS, Radhakrishnan A, Cherian A, Thomas SV (2022). Does etiology and hypsarrhythmia subtype influence outcome in west syndrome? Challenges encountered from a referral center perspective. Neurol India.

[8] Shumiloff NA, Lam WM, Manasco KB (2013). Adrenocorticotropic hormone for the treatment of West Syndrome in children. Ann Pharmacother.

[9] Yamanaka G, Morishita N, Morichi S (2018). Serial analysis of multiple serum cytokine responses to adrenocorticotropic hormone therapy in patients with West syndrome. J Child Neurol.

[10] Ito M, Aiba H, Hashimoto K (2002). Low-dose ACTH therapy for West syndrome: initial effects and long-term outcome. Neurology.

[11] Hamano S, Yamashita S, Tanaka M, Yoshinari S, Minamitani M, Eto Y (2006). Therapeutic efficacy and adverse effects of adrenocorticotropic hormone therapy in west syndrome: differences in dosage of adrenocorticotropic hormone, onset of age, and cause. J Pediatr.

[12] Nakata M, Kato T, Ide M (2016). Long-term weekly ACTH therapy for relapsed West syndrome in tuberous sclerosis complex: a case report. Brain Dev.

[13] Fatema K, Rahman M, Hossain MM, Akhter S, Shomee DA, Akhter S, Mannan M (2021). Pulse methylprednisolone with oral prednisolone versus adrenocorticotropic hormone in children with West syndrome: a randomized controlled trial. J Epilepsy Res.

[14] Munir SZ, Zaman S, McConachie H (1999). Development of an independent behaviour assessment scale for Bangladesh. J Appl Res Intellect Disabil.

[15] Gulati S, Jain P, Kannan L, Sehgal R, Chakrabarty B (2015). The clinical characteristics and treatment response in children with West syndrome in a developing country: a retrospective case record analysis. J Child Neurol.

[16] Kaushik JS, Patra B, Sharma S, Yadav D, Aneja S (2013). Clinical spectrum and treatment outcome of West Syndrome in children from Northern India. Seizure.

[17] Calderón Romero M, Arce Portillo E, López Lobato M, Muñoz Cabello B, Blanco Martínez B, Madruga Garrido M, Alonso Luego O (2018). [Cryptogenic West syndrome: Clinical profile, response to treatment and prognostic factors] [Article in Spanish]. An Pediatr (Engl Ed).

[18] Madaan P, Chand P, Linn K (2020). Management practices for West syndrome in South Asia: a survey study and meta-analysis. Epilepsia Open.

[19] Oguni H, Yanagaki S, Hayashi K, Imai K, Funatsuka M, Kishi T, Osawa M (2006). Extremely low-dose ACTH step-up protocol for West syndrome: maximum therapeutic effect with minimal side effects. Brain Dev.

[20] Kalra V, Gulati S, Pandey RM, Menon S (2001). West syndrome and other infantile epileptic encephalopathies--Indian hospital experience. Brain Dev.

[21] Nikolić DM, Milovanović ID, Medjo BP, Atanasković M, Ivanovski PI, Nikolić AV (2018). Prognostic value of EEG in West syndrome. Vojnosanit Pregl.

[22] Yamada K, Toribe Y, Kimizu T (2014). Predictive value of EEG findings at control of epileptic spasms for seizure relapse in patients with West syndrome. Seizure.

[23] Hayashi Y, Yoshinaga H, Akiyama T, Endoh F, Ohtsuka Y, Kobayashi K (2016). Predictive factors for relapse of epileptic spasms after adrenocorticotropic hormone therapy in West syndrome. Brain Dev.

